# MED13 Gene Mutation Related to Autism Spectrum Disorder: A Case Report

**DOI:** 10.7759/cureus.59904

**Published:** 2024-05-08

**Authors:** Marlene D Rivera, Stephanie N Aponte, Felix Rivera, Norma J Arciniegas, Simón Carlo

**Affiliations:** 1 Biochemistry, Ponce Health Sciences University (PHSU) School of Medicine, Ponce, PRI; 2 Research, Ponce Research Institute, Ponce, PRI; 3 Biochemistry, University of Medicine and Health Sciences (UMHS) School of Medicine, Bassettiere, KNA; 4 Pediatrics, Mayagüez Medical Center, Mayagüez, PRI

**Keywords:** med13-associated syndrome, neurodevelopmental disorders, adhd, mediator complex, asd, med13 gene mutation

## Abstract

This case report highlights an association between the MED13 gene and autism spectrum disorder (ASD). ASD is a neurodevelopmental disorder characterized by impaired social interactions, communication difficulties, and repetitive behaviors. The MED13 gene encodes a subunit of the Mediator complex, which plays a key role in gene expression regulation and transcriptional processes. In this case report, we present a case of a child diagnosed with ASD who underwent whole exome sequencing (WES) and revealed an uncertain heterozygous variant in the MED13 gene. The patient exhibited typical features of ASD, including the following: social and communication deficits, restricted interests, repetitive behaviors, and characteristic dysmorphic facial features. The identification of this MED13 gene variant provides further evidence of its potential involvement in ASD pathogenesis. This case adds to the growing body of evidence linking MED13 gene mutations to ASD susceptibility. Understanding the genetic basis of ASD through case reports can aid in early diagnosis, personalized treatment strategies, and genetic counseling for affected individuals and their families. Further research is warranted to explain the precise mechanisms underlying MED13 gene involvement in ASD.

## Introduction

Autism spectrum disorder (ASD) is a complex neurodevelopmental disorder characterized by impaired social interactions, communication difficulties, and repetitive behaviors. MED13 is a gene that has emerged as a potential candidate in the study of ASD. The MED13 gene encodes a subunit of the Mediator complex, which plays a crucial role in regulating gene expression and transcriptional processes. Studies have indicated a potential link between mutations or variants in the MED13 gene and ASD susceptibility. For instance, a case report described an 800 kb deletion involving the MED13 gene, further supporting its relevance to neurodevelopmental disorders [1]. An additional study involving further delineation of the phenotypic spectrum of pathogenic variants in MED13 presented three families involved in the study, of which the first two were children of similar age with a previously established diagnosis of ASD. Both children underwent WES and were found to have a deletion of two amino acids (p.T292_P293del) located in the N-terminal domain of the MED13 protein and a de novo missense variant (p.Asp358Asn) also located in the N-terminal, respectively [2]. Moreover, mutations in other Mediator complex genes, such as CDK8, MED12, and MEDL13, have been linked to overlapping developmental syndromes, indicating a shared molecular pathway [3].

At present, there is no genetic panel available for diagnosing ASD. However, gaining insights into the impact of the MED13 gene mutation provides valuable information regarding the genetic underpinnings of ASD. Further research could contribute to the development of a genetic panel for diagnosing ASD, facilitating early detection. In addition, such research could aid in developing targeted therapies and personalized interventions for individuals affected by both conditions.

In this case, we report a patient with characteristic dysmorphic facial features, intellectual disability, and impaired social interaction characteristics of ASD who underwent whole-exome sequencing (WES) and was found to have a heterozygous intronic variant of uncertain significance (evidence regarding the complete phenotypic spectrum of this mutation is still in the process of emerging) identified within the MED13 gene.

## Case presentation

An 18-year-old female patient diagnosed with level 3 ASD underwent WES, including copy number variants (CNV) analysis and mitochondrial genome sequencing, to identify any pathogenic or likely pathogenic variants associated with her clinical manifestations and diagnosis. A chromosomal microarray, as recommended by the American College of Medical Genetics (ACMG), had been previously conducted and was found to be normal, prompting the decision to proceed with WES. A heterozygous intronic variant of uncertain significance was identified in the MED13 gene (c. 1009+8A>G). Some clinical manifestations associated with this variant that the patient presented include developmental delay, intellectual disability, expressive speech delay, and language problems. As a newborn, she presented respiratory problems, such as dyspnea, which resulted in a pneumothorax. In addition, she presented food allergies to lactose and soy since birth and to gluten since the age of six, which used to cause her high fevers and constant hospitalizations. However, nowadays consumption of these foods only results in constipation. Carnitine was given during middle childhood (ages 10-12) following a metabolic workup that indicated carnitine deficiency secondary to eating difficulties associated with the sensory disorder related to her autism diagnosis. No major improvements were observed, and carnitine and vitamin supplements were given until levels of carnitine normalized. She presented with poor overall growth and hypotonia.

**Figure 1:**
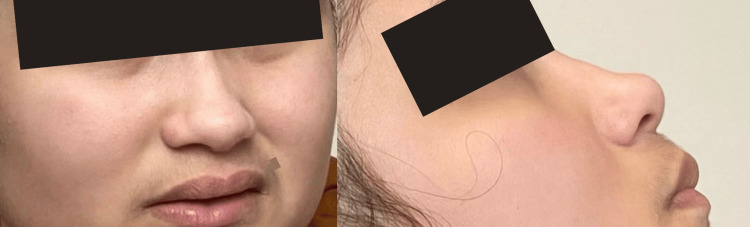
Physical characteristics of the female patient. Her facial features match those reported in the literature: full nasal tip, periorbital fullness, flat philtrum, and synophyrys.

For the evaluation of hypotonia, creatine phosphokinase and aldolase levels were assessed, and they were found to be within normal ranges. Her menarche was at the age of 12. In addition, she suffered from colic and depression and presented sleep disturbances, treated with medication. Through a test of intelligence and cognition, the patient obtained a score below 70, with a diagnosis of severe intellectual disability. She was also diagnosed with receptive language delay, echolalia, speech apraxia, and dysphagia. She uses auto-aggression as a manner to show pain. However, she can follow specific commands, although executing them takes a while. Physical examination revealed synophrys (not shown here as the patient has cosmetic facial hair removal), full nasal tip, flat philtrum, and palate, and a mild protrusion of the upper incisors (Figure 1). She presented with inverted nipples and clitoromegaly and flat and varus feet with calluses on the heels. In addition, she had psoriasis and dermatitis (mainly focused on the scalp) and nail brittleness. Meanwhile, an orthopedic examination revealed crepitus and mild scoliosis, presenting a curvature of less than 20 degrees. The patient also presented severe constipation, requiring a suppository twice a week. At the age of 17, the patient was diagnosed by a psychiatrist with emotional epilepsy, also known as psychogenic nonepileptic seizures (PNES) or psychogenic seizures, for which she is medicated. Other systemic examinations were unremarkable.

## Discussion

**Figure 2:**
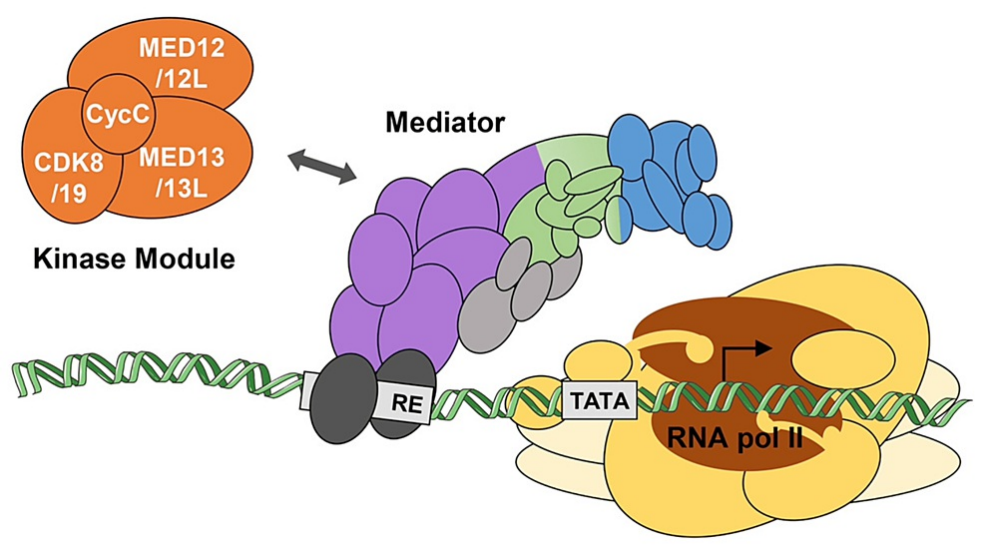
Interaction between the regulatory portion (kinase module), mediator complex, and RNA polymerase Copyright/license: This figure has been adapted from Calpena et al. [6], which is an open-source article distributed under the terms and conditions of the Creative Commons CC-BY license (https://creativecommons.org/licenses/by/4.0/).

The etiology of ASD is composed of environmental and genetic agents. Environmental factors, such as low birth weight, viral infections during pregnancy, exposure to some teratogens, and having older parents, among others, increase the probability of having this disorder [4]. Likewise, more than 100 genes are related to ASD [4]. Among them is the MED13 gene, which was identified in the patient in this case. The chromosomal location of this gene is 17q23.2, which encodes a 240 kDa protein [5]. MED13 is part of the Mediator complex’s regulatory portion, which comprises 26 subunits. These multiple proteins function as a bridge that communicates transcription factors bound to DNA with the RNA polymerase II, hence its role in the regulation of gene expression [6]. The regulatory portion can be reversibly associated with the Mediator complex (Figure 2). This portion is composed of a four-subunit kinase, also known as a module, in which cyclin C (CycC) is combined with cyclin-dependent kinase 8 (CDK8), MED12, and MED13 or with its respective paralogs cyclin-dependent kinase 19 (CDK19), MED12L and MED13L [4]. The CDK8 module, of which MED13 is a part, can function as a transcription activator or repressor [7]. Moreover, MED13 binds to nuclear thyroid hormone receptors and vitamin D receptors [2].

Due to the interaction of MED13 with CDK8, MED12, and their paralogs, mutations in these genes have been shown to cause similar phenotypes, such as developmental delay, speech delay, visual abnormalities, and congenital heart defects, which in some literature have been described as a module-related syndrome [3,6]. In addition, there is little information related to the MED13 gene compared to the rest of the genes, which makes it difficult to distinguish the different syndromes [3]. For this reason, the purpose of this case report is to provide more information related to MED13-associated syndrome.

In a study of 13 patients with a MED13 mutation, 11 had a de novo mutation. The mutation was inherited from mother to child in the other two cases. Other studies suggest that incomplete maternal penetrance may occur [2]. The most common MED13 mutations observed include nonsense, missense, and frameshift mutations, typically located at the N-terminal of the protein. However, no correlation has been identified between any of these mutation types and the presentation or phenotypic severity [8].

The MED13-associated syndrome is linked to different neurodevelopmental disorders. Mainly, it is associated with delays in speech and language, both expressive and receptive, ranging from moderate to severe [8], and ASD and attention-deficit/hyperactivity disorder (ADHD). In general, most patients present developmental delays with intellectual disability ranging from borderline to moderate and poor body growth [2]. Likewise, of the 13 patients in the study, seven presented delayed motor development [8]. Other conditions common to this syndrome are ocular and visual anomalies, such as strabismus, nystagmus, Duane's anomaly, and astigmatism [8]. In addition, they may suffer from severe constipation. Other conditions related to this syndrome are congenital heart defects, conductive hearing loss, hypotonia, and sleep problems. The most common facial features are a high anterior hairline, broad and high nasal bridge, full nasal tip, flat philtrum, wide mouth with thin upper lip, periorbital fullness, short palpebral fissures, and synophrys [2,8].

Unlike the mutations reported in the literature, the patient's mutation is in an intron. Our case provides information about another way this gene can be mutated. Neither parent had symptoms related to MED13-associated syndrome, nor were they tested for mutation carriers. Therefore, based on the autosomal dominant inheritance pattern, it can be inferred that the mutation is likely de novo. Other studies have suggested the possibility of incomplete penetrance by the mother [2]. Like other cases reported, the patient presents severe speech and language problems, constipation, and sleep disorders. However, the patient presents echolalia, which other articles about this gene do not mention. The patient's intellectual disability is severe, compared to the range reported in other studies that go from borderline to moderate. Her facial features match those reported: full nasal tip, periorbital fullness, flat philtrum, and synophrys [2]. Other clinically relevant conditions including scoliosis, flat palate, jaw prognathism, dermatitis, psoriasis, multiple allergies, flat feet, varus feet, clitoral enlargement, and introverted nipples are not described in other studies related to this gene.

The current treatment for this syndrome is occupational, speech, and psychological therapy. In addition, medications to control other symptoms, such as sleep disorders and constipation, have been helpful.

## Conclusions

In this study, we present the case of an 18-year-old female patient harboring a heterozygous intronic variant of uncertain significance in the MED13 gene (c.1009+8A>G), exhibiting an autosomal dominant inheritance pattern. Variants in MED13 are associated with neurodevelopmental disorders, such as ASD, intellectual disability, speech delay, and mild facial dimorphisms, characteristics that our patient presented. Therefore, our study supports the role of MED13 in the neurodevelopmental process and encourages clinical researchers to conduct further studies to understand the genetic bases of ASD and, consequently, provide early diagnosis, elaborate personalized treatment strategies, and promote genetic counseling for affected individuals and their families.
